# Induced Resistance Against Western Flower Thrips by the *Pseudomonas syringae*-Derived Defense Elicitors in Tomato

**DOI:** 10.3389/fpls.2018.01417

**Published:** 2018-09-26

**Authors:** Gang Chen, Rocío Escobar-Bravo, Hye Kyong Kim, Kirsten A. Leiss, Peter G. L. Klinkhamer

**Affiliations:** ^1^Plant Science and Natural Products, Institute of Biology, Leiden University, Leiden, Netherlands; ^2^Business Unit Horticulture, Wageningen University and Research Center, Bleiswijk, Netherlands

**Keywords:** coronatine, *Frankliniella occidentalis*, induced plant defenses, jasmonic acid, *Pseudomonas syringae*, salicylic acid, *Solanum lycopersicum*, type VI glandular trichomes

## Abstract

Western flower thrips (WFT) *Frankliniella occidentalis* (Pergande) is a key agricultural pest of cultivated tomatoes. Induced host plant resistance by activating jasmonic acid (JA) signaling pathway constitutes a promising method for WFT control. The phytotoxin coronatine (COR), produced by *Pseudomonas syringae* pv. tomato DC3000 (*Pst*), mimics the plant hormone JA-Isoleucine and can promote resistance against herbivorous arthropods. Here we determined the effect of *Pst* and COR on tomato resistance against WFT, induction of JA and salicylic acid (SA) associated defenses, and plant chemistry. Additionally, we investigated the presence of other components in *Pst*-derived and filtered culture medium, and their interactive effect with COR on tomato resistance to WFT. Our results showed that infiltration of COR or *Pst* reduced WFT feeding damage in tomato plants. COR and *Pst* induced the expression of JA-associated gene and protein marker. COR also induced expression of a SA-related responsive gene, although at much less magnitude. Activation of JA defenses in COR and *Pst* infiltrated plants did not affect density of type VI leaf trichomes, which are defenses reported to be induced by JA. An untargeted metabolomic analysis showed that both treatments induced strong changes in infiltrated leaves, but leaf responses to COR or *Pst* slightly differed. Application of the *Pst*-derived and filtered culture medium, containing COR but not viable *Pst*, also increased tomato resistance against WFT confirming that the induction of tomato defenses does not require a living *Pst* population to be present in the plant. Infiltration of tomato plants with low concentrations of COR in diluted *Pst*-derived and filtered culture medium reduced WFT feeding damage in a greater magnitude than infiltration with an equivalent amount of pure COR indicating that other elicitors are present in the medium. This was confirmed by the fact that the medium from a COR-mutant of *Pst* also strongly reduced silver damage. In conclusion, our results indicate that induction of JA defenses by COR, *Pst* infection, the medium of *Pst* and the medium of a *Pst* COR^-^ mutant increased resistance against WFT. This was not mediated by the reinforcement of leaf trichome densities, but rather the induction of chemical defenses.

## Introduction

The western flower thrips (WFT), *Frankliniella occidentalis* [Pergande], is one of the most serious greenhouse and field insect pests of vegetable and ornamental crops worldwide. It is a highly polyphagous insect that can feed on more than 200 wild and cultivated host species (Lewis, 1997) by piercing and sucking epidermal/mesophyll plant cells, which results in damaged areas of a silvery appearance (silver damage). WFT cause direct damage by feeding on leaves, flowers, and fruits, or indirect damage through the transmission of plant viruses (de Jager et al., 1995a,b), being the main vector of tospoviruses, such as tomato spotted wilt virus (Maris et al., 2003). Current control of WFT mainly depends on the use of pesticides and biological control. Use of pesticides leads to residue problems on marketable crops, human health risks, toxicity to non-target beneficial organisms, and environmental contamination (Bielza, 2008; Demirozer et al., 2012; Gao et al., 2012; Mouden et al., 2017). Therefore, multiple and complementary tactics are necessary in the framework of integrated pest management (IPM) programs.

Enhancement of constitutive and/or inducible host plant defenses against WFT has recently been discussed as a promising alternative for thrips control (Steenbergen et al., 2018). Plants defend themselves against herbivores by employing a plethora of physical and chemical arsenals, including trichomes, defensive enzymes, and secondary metabolites that can be present in the plant before attack or induced after detecting the presence of the attacker. Induced plant defenses against herbivory are mainly controlled by the phytohormones jasmonic acid (JA), salicylic acid (SA), and ethylene (ET), and fine-tuned by other phytohormones such as abscisic acid, auxins, cytokinins, and gibberellins (Pieterse et al., 2012). Activation of JA-associated defenses has been reported to confer plant resistance against pierce-sucking arthropods such as spider mites and thrips (Li et al., 2002; Ament et al., 2004). In particular, WFT have been reported to be susceptible to JA-associated induced defenses in diverse plant species such as *Arabidopsis*, Chinese cabbage (*Brassica rapa*), cotton (*Aphis gossypii*), and tomato (*Solanum lycopersicum*) (Omer et al., 2001; Abe et al., 2008; Abe et al., 2009; Escobar-Bravo et al., 2017).

In tomato (*S. lycopersicum*), induction of JA-related defenses has been associated to increased levels of defensive type-VI glandular trichomes and their derived exudates, proteins such as proteinase inhibitors and polyphenol oxidases (PPO), and secondary metabolites (Boughton et al., 2005; Degenhardt et al., 2010; Escobar-Bravo et al., 2017). Type-VI trichomes are important physical and chemical defense barriers, and their absence increases tomato susceptibility against herbivory (Kang et al., 2010a,b). Overexpression of certain proteinase inhibitors has been reported to increase plant resistance against WFT (Annadana et al., 2002; Outchkourov et al., 2004), and enhanced PPO activities can confer enhanced resistance against beet armyworm (*Spodoptera exigua*), cotton bollworm (*Helicoverpa armigera*) (Bhonwong et al., 2009), and cutworm (*Spodoptera litura*) (Mahanil et al., 2008). Accordingly, application of natural or synthetic elicitors activating these JA-associated defenses can increase tomato resistance against various insects, including WFT (Thaler, 1999; Thaler et al., 2002; Escobar-Bravo et al., 2017).

The phytotoxin coronatine (COR) produced by several pathovars of *Pseudomonas syringae* acts as a virulence factor in *P. syringae* pv. tomato (*Pst*), allowing this pathogen to successfully develop high populations in the plant (Zhao et al., 2001; Uppalapati and Bender, 2005; Zheng et al., 2012). COR is a polyketide formed by the coupling of coronafacic acid (CFA) and coronamic acid (CMA) through an amide bond (Bender et al., 1993). Its structure mimics a bioactive JA conjugate (JA-Isoleucine), thus having the ability to stimulate JA-associated defense responses (reviewed by Geng et al., 2014), but also affecting ET and auxin signaling pathways (Uppalapati et al., 2005). Both JA and COR can induce chlorosis, ET emission, inhibition of root elongation, volatile production, biosynthesis of stress-associated compounds and anti-herbivore proteins (Uppalapati et al., 2005). Consequently, infiltration with COR-producing *P. syringae* or infiltration of pure COR in *Arabidopsis* enhanced plant resistance against arthropod herbivores, such as the caterpillar *Trichoplusia ni* (Cui et al., 2005). In tomato, *P. syringae* infection (López-Gresa et al., 2011) or COR application (Uppalapati et al., 2005) also induces metabolomic changes in the plant. All these studies suggest that *Pst* may potentially be used to increase resistance against WFT in tomato. Yet, the effects of *Pst* and COR infiltration on tomato defenses against herbivory may differ. Activation of defense signaling pathways in *Pst*-infected plants is not only mediated by the phytotoxin COR, but also by an array of virulence factors such as exopolysaccharides effectors secreted by the type III secretion system, and cell-wall-degrading enzymes (Zhao et al., 2003; He et al., 2004). Thus, research on the possible use of other *Pst-*derived defense elicitors for their practical application in agricultural systems is crucial, as *Pst* is a plant pathogen.

Here, we first investigated the effect of COR or *Pst* DC3000 infiltration on tomato defenses against WFT. In particular, we determined their effect on WFT-associated feeding damage, as well as variations in type-VI leaf trichome densities, leaf metabolome, expression of defense-associated genes, and tomato growth. In a further attempt to test the possible role of other *Pst* DC3000 associated defense elicitors in tomato-WFT interaction, we also studied the effect of dilution series of the *Pst*-derived filtered medium and a COR-deficient *Pst* strain on tomato resistance against WFT and activation of JA signaling pathway.

## Materials and Methods

### Plants, Insect, and Bacterial Strains

The tomato cultivar “Moneymaker” (*S. lycopersicum*) was used in all experiments. Tomato seeds were germinated on filter paper soaked with MilliQ water and incubated at 20°C. Five days later, germinated seeds were planted in plastic pots (11 cm × 11 cm × 12 cm) filled with potting soil and maintained in a climate room at 20°C, 70% RH, 113.6 μmol photons m^-2^⋅s^-1^ of photosynthetically active radiation (PAR) and L16:D8 photoperiod.

Western flower thrips, *F. occidentalis* (Pergande), were maintained on chrysanthemum flowers (cultivar Euro Sunny) in a climate room at 23°C, 60% RH, and L12:D12 photoperiod.

*Pseudomonas syringae* pv. *tomato* DC3000 (*Pst* DC3000) NCPPB4369 was obtained from the National Collection of Plant Pathogenic Bacteria (NCPPB, London, United Kingdom). *P. syringae* pv. *tomato* DB29 (*Pst* DB29, a *cmaA cfaA* double mutant of *Pst* DC3000) (Brooks et al., 2004) was kindly provided by Prof. Barbara Kunkel from Washington University in St. Louis. Both *Pst* DC3000 and *Pst* DB29 were stored in 30% glycerol at -80°C for long-term preservation.

### Experimental Design

To determine the effect of COR and *Pst* DC3000 on tomato defenses against WFT (Experiment 1), 4-week-old tomato plants were infiltrated with: (1) 5 μM COR solution, (2) a 10^8^ cfu ml^-1^ of *Pst* DC3000 suspension, or (3) a mock solution of sterilized MilliQ water. For this, four leaflets (two top leaflets of leaf 2 and 3 from the bottom) were pressure-infiltrated with 400 μl (100 μl for each leaflet) of one of the treatments on their abaxial leaf sides using a 1-ml needleless syringe. Seven days after infiltration, plants were sampled for type VI trichome density, metabolomics, gene expression, and polyphenol oxidase (PPO) activity analysis, or used for non-choice whole plant thrips bioassays.

With the aim to explore whether COR and other defense elicitors present in *Pst* DC3000-derived medium (without viable bacteria) increases tomato resistance against WFT, three additional experiments were conducted. First, to test if the COR present in *Pst* DC3000-derived medium can enhance tomato resistance against WFT (Experiment 2), tomato plants at four leaf-stage were infiltrated with 100 μl of (1) mock solution (MilliQ water), (2) blank medium (described in Generation of *Pst-*derived and Blank Medium below), (3) blank medium supplemented with 0.68 μM COR (blank medium + COR), (4) 10^8^ cfu ml^-1^ of *Pst* DC3000 suspension (*Pst* DC3000), or (5) *Pst* DC3000*-*derived medium (*Pst* DC3000 medium, without viable bacteria) containing 0.68 μM COR. The COR in the *Pst* DC3000*-*derived medium was produced by the bacteria during 6-day cultivation, and the concentration was measured before the start of the experiment. Second, to test the existence of interactions of COR with other defense elicitors present in *Pst* DC3000-derived medium on tomato resistance against WFT (Experiment 3), 4-week-old tomato plants were infiltrated with 0.2×, 0.4×, 0.6×, 0.8×, and 1.0× concentrations of (1) blank medium, (2) 0.68 μM COR diluted with blank medium, or (3) *Pst* DC3000-derived medium containing 0.68 μM COR. Third, to confirm the effect of other defense elicitors, present in *Pst* DC3000-derived medium, on tomato resistance against WFT (Experiment 4), 4-week-old tomato plants were infiltrated with (1) blank medium, (2) 0.14 μM COR diluted with blank medium, (3) culture medium derived from *Pst* DB29, a COR^-^ mutant bacteria of *Pst* DC3000, diluted five fold with blank medium, or (4) five fold diluted *Pst* DB29*-*derived medium containing 0.14 μM COR. In these three experiments, four leaflets (two top leaflets of each of leaves 2 and 3 from the bottom) from one tomato plant were pressure-infiltrated on their abaxial leaf sides with about 400 μl in total of corresponding treatments as described above. Seven days after infiltration, parts of the plants were sampled for PPO activity measurements and the other part was used for non-choice whole plant thrips bioassays.

### *Pst* Cultivation and Suspension Preparation

*Pst* DC3000 and *Pst* DB29 were cultured in a King’s B medium plate (King et al., 1954) supplemented with 100 μg ml^-1^ rifampicin and grown for 2 days at 28°C prior to use (Katagiri et al., 2002). The activated *P. syringae* pv. *tomato* (*Pst*) was then transferred to King’s B liquid medium supplemented with 100 μg ml^-1^ rifampicin in a shaking incubator (200 rpm) at 28°C for 8 to 12 h (Katagiri et al., 2002).

To prepare *Pst* DC3000 suspension, the obtained *Pst* DC3000 King’s B liquid culture was centrifuged at 4,000 rpm for 10 min at 4°C. The supernatant was discarded, and the bacteria pellet was re-suspended in sterilized MilliQ water. The bacteria suspension was diluted with sterilized MilliQ water to reach a concentration of 10^8^ colony-forming units (cfu) ml^-1^, estimated by an optical density at 600 nm of 0.5, which was used for the experiments.

### Generation of *Pst-*Derived and Blank Medium

*Pst*-DC3000- and *Pst-*DB29-derived medium were obtained following the protocol of Palmer and Bender (1993) with some modifications. Briefly, 100 μl of the *Pst* King’s B liquid culture obtained as described above was added to 20 ml Hoitink and Sinden medium optimized for COR production (also known as HSC) (nutrients per liter with a pH = 6.8: 1.0 g NH_4_Cl, 0.2 g MgSO_4_⋅7H_2_O, 4.1 g KH_2_PO_4_, 3.6 g K_2_HPO_4_⋅3H_2_O, 0.3 g KNO_3_, 20 μM FeCl_3_, 20 g glucose) supplemented with 100 μg ml^-1^ rifampicin and grown in a shaking incubator (200 rpm) at 20°C for 6 days. The *Pst* culture (20 ml) was centrifuged at 4,000 rpm for 30 min at 4°C. The supernatants were filtered using 0.22 μm regenerated cellulose (RC) filters (Sartorius AG, Göttingen, Germany) to remove *Pst* from the medium. The absence of active bacteria in the *Pst*-derived medium was confirmed by spraying the filtered *Pst-*derived medium on plates of King’s B medium and culturing the plates at room temperature. No colonies were detected at 3 days after the initial culture. Blank medium used as a control in Experiment 2, 3, and 4 was generated by incubating fresh HSC medium supplemented with 100 μg ml^-1^ rifampicin in a shaking incubator (200 rpm) at 20°C for 6 days. Thereafter, the HSC culture was centrifuged and the resulting supernatant was filtered using 0.22 μm RC filters. Presence of bacteria in the blank medium was also checked as described above for *Pst-*derived medium.

### Measurements of Coronatine Concentration in *Pst* DC3000*-*Derived Medium

Concentration of COR in the *Pst* DC3000-derived medium was determined by HPLC as described by Palmer and Bender (1993) with slight modifications. In short, 20 ml of *Pst* DC3000-derived filtered medium was adjusted to pH = 9 and extracted twice with 20 ml of ethyl acetate. The aqueous phase was adjusted to pH = 2 and then extracted three times with 20 ml ethyl acetate. The ethyl acetate phase was dried in 45°C water bath in 50 ml tubes. The COR was recovered by re-dissolving twice with 250 μM 20% acetonitrile. Three samples were analyzed on a reverse-phase C-8 column (150 mm × 4.6 mm, 5 μm particle size, Agilent Zorbax Eclipse XDB) at 208 nm. The mobile phases, A and B, were MilliQ water and HPLC-grade acetonitrile, respectively. The flow rate was kept constant at 1 ml/min. The gradient elution was as follows: 0.00 min at 80% A, 5.00 min at 35% A, 7.00 min/10% A, 8.00 min/10% A, 8.10 min/80% A, 10.00 min/80% A. The injection volume was 50 μl, and the column temperature was 25°C. Calibration curves for quantification of COR were constructed by using dilution series of commercially available COR (Sigma-Aldrich, St. Louis, MO, United States).

### Growth of *Pst* DC3000 in Infiltrated Tomato Leaves

Bacteria growth in the leaflets of *Pst* DC3000*-*infiltrated plants was confirmed at 7 days after infiltration. For this, one of the *Pst* DC3000*-*infiltrated leaflets was surface sterilized by placing it in a 70% ethanol solution for 1 min, blotted briefly on paper towels and rinsed in sterile distilled water for 1 min. Thereafter, a leaf disk (1.5 cm diameter) was punched and placed in a 3-ml microfuge tube with 100 μl sterile distilled water. The samples were ground and subsequently vortexed for 10 s; 10 μl of the leaf disk solution was plated on King’s B medium supplemented with 100 μg ml^-1^ rifampicin and incubated at room temperature. Number of cfu was recorded at 3 days after incubation.

### Non-choice Whole Plant Thrips Bioassay

A non-choice whole plant thrips bioassay was used to test tomato resistance against WFT (Leiss et al., 2009b). For this, plants were placed inside individual WFT-proof cages consisting of transparent plastic cylinders (50 cm height, 20 cm diameter), closed at the top with displaceable lids made of nylon gauze of 120 μm mesh size. Ten adult WFT (eight females and two males) were released into each cage. Plants were maintained in a climate room with 113.6 μmol photons m^-2^⋅s^-1^ of PAR, 16L:8D of photoperiod, 25°C and 70% RH for 7 days. WFT feeding damage (hereafter referred as “silver damage”) was evaluated in all the leaves of the plant 7 days after infestation, and expressed as the damaged area in millimeter square. Evaluation of WFT-associated leaf damage in the whole plant has been proved to correlate well with resistance-associated parameters such as number of larvae, adult survival, adult abundance, and preference (De Kogel et al., 1997; Jiang et al., 2005; Badenes-Pérez and López-Pérez, 2018), and it has been used in multitude of studies determining host plant resistance to WFT (e.g., Leiss et al., 2009a,b, 2013; Mirnezhad et al., 2010; Thoen et al., 2016; Escobar-Bravo et al., 2017; Badenes-Pérez and López-Pérez, 2018; Escobar-Bravo et al., in press^1^). Silver damage symptoms caused by infestation with 10 adult WFT were very subtle at 7 days after infestation, and it did not result in significant loss of leaf tissues (see **Supplementary Figure S1**). Thus, WFT development and feeding was not limited by the available plant material in the host plant.

### Measurement of PPO Activity

Polyphenol oxidase (PPO) activity was measured in one of the infiltrated leaflets belonging to the second leaf from the bottom using the protocol described in Stout et al. (1998). In short, 0.150 g of frozen and ground plant material was homogenized in a 2 ml tube with 1.25 ml ice-cold 0.1 M pH 7.0 potassium phosphate buffer containing 7% polyvinyl polypyrolidine and 0.4 ml of 10% Triton X-100. The extracts were vortexed for 2 min and centrifuged at 11,000 × *g* for 10 min at 4°C. Five microliters of the enzyme extract were added to 1 ml of 2.92 mM chlorogenic acid solution in pH 8.0 potassium phosphate buffer. The optical density (OD) at 470 nm was recorded in a spectrophotometer (UV-1800, Shimadzu) every 10 s for 1 min. PPO activity was expressed as changes in OD values per min per gram of fresh weight.

### Gene Expression Analysis by RT-qPCR

Expression of the JA- and SA-associated marker genes, the *wound-inducible proteinase inhibitor II* (*WIPI-II*, also known as *PI-II)* and the *pathogenesis-related protein 6* (*PR-P6*, also known as *PR-1b*) (Alba et al., 2015), respectively, were determined in mock-, COR-, and *Pst* DC3000-treated plants at 7 days after infiltration. The two infiltrated leaflets of leaf 3 from the bottom were flash frozen, homogenized, and stored at -80°C until analysis. Around 100 mg of the leaf material was used for RNA isolation. Total RNA was extracted using a phenol/LiCl method (Verdonk et al., 2003) followed by DNase (Ambion) treatment. Single strand cDNA was synthesized from 4 μg total RNA in a 20-μl reaction using a M-MuLV Reverse Transcriptase (Fermentas) according to manufacturer’s recommendations. The quantity of targeted synthesized cDNAs was analyzed with real-time quantitative reverse transcription-PCR (qRT-PCR) in CFX96^TM^ Optics Module (BIO-RAD) using iQ^TM^ SYBR Green Supermix (BIO-RAD). The PCR protocol was set up in 20 μl reactions containing 0.25 μM of each primer and 1 μl of cDNA. The PCR program was as follows: 50°C for 5 min, 95°C for 2 min, 40 cycles of 95°C for 15 s, 60°C for 1 min, followed by a melting curve analysis. Four biological replicates (i.e., individual plants) for each treatment were used for qRT-PCR analysis and two technical replicates were analyzed per treatment. *Actin* was used as internal standard for the normalization of expression levels for both targeted genes. The normalized expression (NE) of both genes was calculated using the 2^-ΔΔCt^ method (Livak and Schmittgen, 2001). To illustrate the levels of gene expressions in plot, NE values were scaled to the treatment with the lowest average NE, which was set to 1. The gene specific qRT-PCR primers are shown in **Supplementary Method S1**.

### Trichome Density Measurement

Type-VI glandular trichome density was determined on non-infiltrated leaflets of mock-, COR-, and *Pst* DC3000*-*treated plants at 7 days after infiltration. For this, the second terminal leaflet of the third leaf from the bottom was used. Two pictures were taken in the middle section of the leaflet, at both sides of the midrib, in the adaxial and abaxial leaf sides by using a Leica stereomicroscope (MZ16, Leica Microsystems, Wetzlar, Germany). Each picture corresponded to an area of 12 mm^2^. Trichome number was counted on the pictures using the software 64-bit Fiji ImageJ^2^. The average of these two measurements was calculated for each leaflet and expressed as number of type-VI trichomes per centimeter square.

### Nuclear Magnetic Resonance (NMR) Analysis

NMR metabolomic analysis was performed on mock-, COR-, or *Pst* DC3000*-*infiltrated leaflets at 7 days after infiltration. For this, plant material was freeze-dried and ground using a tissue lyser (Qiagen, Hilden, Germany). Twenty milligrams of fine powder were extracted with 1.5 ml of 80% methanol-*d4* in KH_2_PO_4_ buffer (90 mM, pH = 6.0) containing 0.02% (w/v) trimethyl silyl-3-propionic acid sodium salt-*d4* (TMSP). Plant extracts were vortexed for 1 min, ultra-sonicated for 15 min, and centrifuged at 13,000 rpm for 15 min at room temperature. Eight hundred microliters of the supernatant was transferred to the NMR tubes for analysis.

The ^1^H NMR spectra were acquired using a 600-MHz Bruker AV-600 spectrometer equipped with cryo-probe operating at a proton NMR frequency of 600 MHz at 25°C, as described in López-Gresa et al. (2012). Deuterated methanol served as internal lock. Each ^1^H NMR spectrum consisted of 128 scans requiring 10 min acquisition time with a digital resolution of 0.25 Hz/point, a pulse angle of 30° (10.8 μs), and a recycle delay of 1.5 s per scan. A pre-saturation sequence was used to suppress the residual water signal with low power selective irradiation at the H_2_O frequency during the recycle delay. Spectra were Fourier transformed with a 0.3-HZ line broadening and zero-filled to 32 K points. Phase and baseline correction of the resulting spectra were done manually, followed by a calibration to TMSP at 0.00 ppm using Topspin (version 2.1, Bruker). ^1^H NMR spectra were then converted and saved as ASCII files using AMIX (v. 3.7, Bruker Biospin). Spectral intensities were scaled to the intensity of the internal standard TMSP and reduced to integrated regions, referred to as buckets, of equal width (0.04 ppm) corresponding to the region of δ 10.0–0.2. The regions in the range of δ 4.92–4.70 and δ 3.33–3.28, corresponding to water and methanol, respectively, were removed prior to statistical analyses.

### Statistical Analysis

All statistical analyses were performed using the SPSS software package (version 23; SPSS Inc., Chicago, IL, United States). Effect of mock-solution, COR, *Pst* DC3000, or *Pst* DC3000-derived medium infiltration on silver damage symptoms, type VI trichome density, PPO activity, and normalized expression of *WIPI-II* and *PR-P6* (Experiments 1 and 2) were analyzed by one-way ANOVA, followed by Fisher’s least significant difference (LSD) *post hoc* test. Residuals of the data were first tested for normality and homogeneity of variance. Data on silver damage and *WIPI-II* and *PR-P6* expression obtained from Experiment 1 and PPO activity determined in Experiment 2 were Log transformed prior to analysis to meet ANOVA assumptions. Effect of treatments (blank medium, blank medium + COR, and *Pst* DC3000-derived medium), concentration (0.2, 0.4, 0.6, 0.8, and 1.0×), and the interaction between these two factors (Experiment 3) on silver damage symptoms, and PPO activity was determined by generalized linear models (GLM) using linear distribution and identity link functions, followed by LSD *post hoc* test. Data on silver damage were Log-transformed prior to analysis. Effect of COR, *Pst* DB29-derived medium, and their interaction (Experiment 4) on silver damage and PPO activity was analyzed by GLM using linear distribution and identity link functions, followed by LSD *post hoc* test. Data on silver damage were Log-transformed prior to analysis. Patterns of chemical shifts detected by NMR in leaf extracts of mock-, COR-, or *Pst* DC3000-treated plants were subjected to multivariate analysis using the SIMCA-P 15 software package (Umetrics, Umeå, Sweden). Supervised partial least squares discriminant analysis (PLS-DA) was applied to determine the variation in X variables (chemical shifts) modeled by the Y explanatory variable corresponding to mock, COR or *Pst* DC3000 treatments. *R^2^X* and *R^2^Y* is the cumulative variation explained by the PLS-DA model in variable X and Y, respectively. *Q^2^* is the cumulative predicted variation in Y, according to cross-validation. The final model was the one with minimum number of latent variables showing the highest value of *Q^2^*. The chemical shifts with a variable importance in projection (VIP) > 1 were selected as the important X variables, some of which were identified and tested using a nonparametric analysis followed by non-parametric Kruskal–Wallis test. Detailed statistical results are shown in **Supplementary Table S1**.

## Results

### Infiltration of COR or *Pst* DC3000 Increases Tomato Resistance Against WFT

**FIGURE 1:**
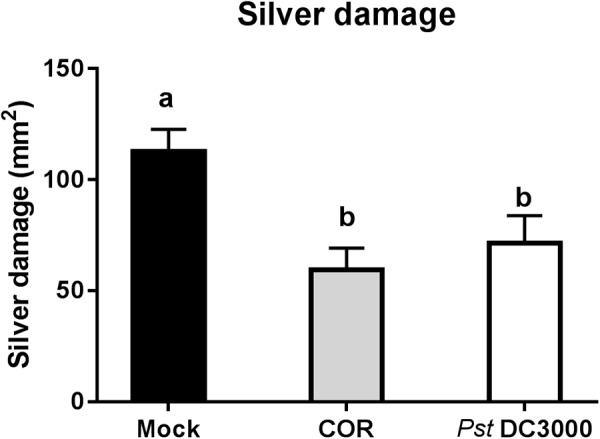
Effect of COR and *Pst* DC3000 on tomato resistance against WFT. Silver damage symptoms (mean ± SEM, *n* = 15) in tomato plants infiltrated with mock solution (mock), coronatine (COR), or *Pseudomonas syringae* pv. tomato DC3000 (*Pst* DC3000). Plants were infested with western flower thrips (WFT) at 7 days after the initial treatment and evaluated 7 days after WFT infestation. Different letters indicate significant differences among treatments tested by Fisher’s LSD test at *P* < 0.05.

Infiltration of tomato plants with COR or *Pst* DC3000 reduced silver damage by 47% and 37%, respectively, compared to the mock-treated plants (ANOVA: *P* < 0.05, **Figure 1**). Overall, this reduction was evident in both infiltrated and non-infiltrated leaves of COR- and *Pst* DC3000-treated plants (ANOVA: *P* < 0.05, **Supplementary Figure S2**).

### COR and *Pst* DC3000 Induced JA-Signaling, and COR Induced Both JA and SA

**FIGURE 2:**
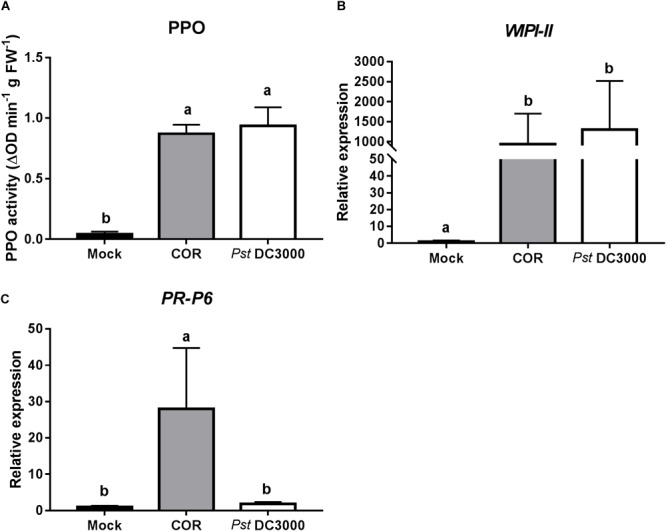
Effect of COR and *Pst* DC3000 on jasmonic acid- and salicylic acid-associated responses. **(A)** Polyphenol oxidase (PPO) activity (mean ± SEM, *n* = 5) and relative transcript levels of **(B)** the JA-responsive gene *wound inducible proteinase inhibitor-II* (*WIPI-II*) and **(C)** the SA-responsive gene *pathogenesis related protein 6* (*PR-P6*) (mean ± SEM, *n* = 5) were measured in tomato plants at 7 days after infiltration with coronatine (COR), *Pseudomonas syringae* pv. tomato DC3000 (*Pst* DC3000), or a mock solution (mock). The analysis was performed on infiltrated leaflets from the bottom second/third leaf. Different letters indicate significant differences among treatments tested by Fisher’s LSD test at *P* < 0.05.

To further determine the mechanism of COR and *Pst* DC3000*-*mediated induction of tomato defenses against WFT, expression of JA- and SA-responsive genes, as well as the activity of the JA-associated defensive protein PPO, were analyzed at 7 days after infiltration. Both COR and *Pst* DC3000 infiltration strongly induced PPO activity in infiltrated tomato leaves (ANOVA: *P* < 0.05, **Figure 2A**). Similarly, the expression of *WIPI-II*, a JA marker gene, was about 900 and 1,300 times higher in COR- and *Pst* DC3000-infiltrated plants, respectively, than in mock-treated leaves of control plants (ANOVA: *P* < 0.05, **Figure 2B**). Interestingly, for *PR-P6*, a SA marker gene, a 28-times higher expression was observed in COR-treated plants, but not in mock and *Pst* DC3000-infiltrated plants (ANOVA: *P* < 0.05, **Figure 2C**).

### Infiltration of Tomato Plants With COR or *Pst* DC3000 Does Not Increase Type-VI Trichome Density

**FIGURE 3:**
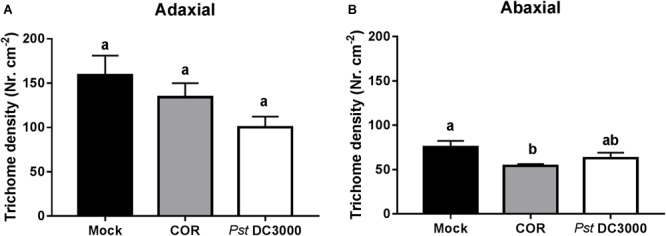
Effect of COR and *Pst* DC3000 on type VI trichome density. Type VI trichome density (mean ± SEM, *n* = 10) on **(A)** adaxial or **(B)** abaxial leaf side was determined in tomato plants infiltrated with coronatine (COR), *Pseudomonas syringae* pv. tomato DC3000 (*Pst* DC3000), or a mock solution (mock) at 7 days after the initial treatments. The analysis was performed in leaflets collected from the third youngest leaf. Different letters indicate significant differences among treatments tested by Fisher’s LSD test at *P* < 0.05.

To determine whether COR and *Pst* DC3000 induce trichome-associated defenses against WFT, type-VI trichome density was determined on both adaxial and abaxial leaf sides of mock-, COR-, and *Pst* DC3000*-*treated plants. Surprisingly, type-VI trichome density in the adaxial leaf side was marginally decreased by COR or *Pst* DC3000 infiltration (ANOVA: *P* = 0.071, **Figure 3A**). However, type-VI trichome density on abaxial leaf sides was slightly reduced in COR-treated plants in comparison to *Pst* DC3000- and mock-treated plants (ANOVA: *P* < 0.05, **Figure 3B**).

### COR and *Pst* DC3000 Induced Similar Metabolomic Changes in Infiltrated Tomato Leaves

**FIGURE 4:**
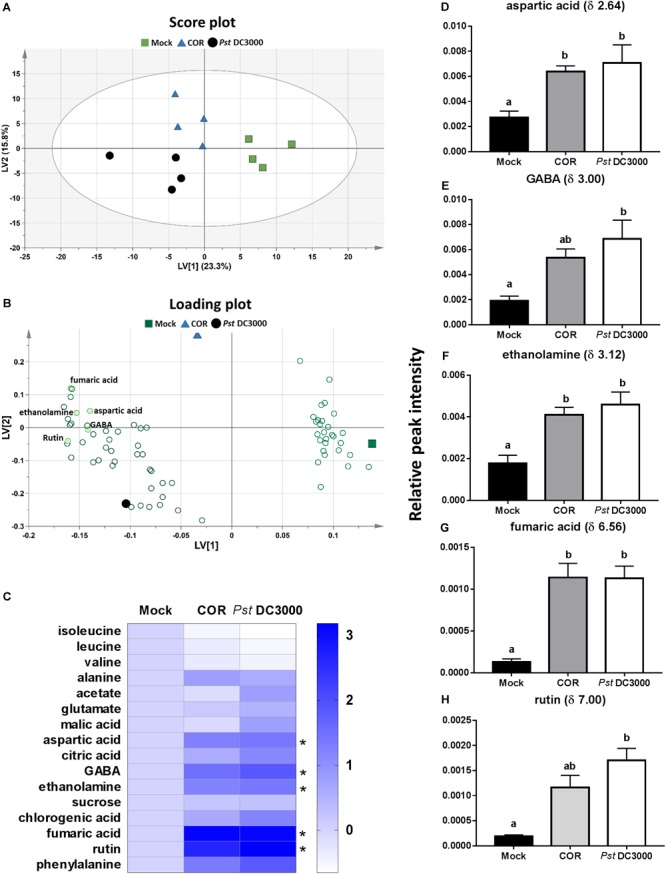
Metabolome responses of tomato plants to COR and *Pst* DC3000 infiltration. Leaf metabolites were analyzed on tomato leaves infiltrated with coronatine (COR), *Pseudomonas syringae* pv. tomato DC3000 (*Pst* DC3000), or a mock solution (mock) by NMR at 7 days after the initial treatment. Partial least square-discriminant analysis (PLS-DA) was performed based on ^1^H-NMR spectra (*n* = 4 individual plants), and resulted in five latent variables (LVs) that cumulatively explained 74.6% of the total metabolomic variation and 91.1% of the treatment response, with a 40.7% total model predictability. **(A)** Score plot showing the first two LVs. The ellipse represents the Hotelling T2 with 95% confidence in score plot. **(B)** Loading plot showing important metabolites contributing most to the model (VIP score > 1). **(C)** Heatmap of the identified 16 compounds. Each of the three Heatmap columns represents the log_2_ fold change of relative peak intensity from one of the treatments Mock, COR, or *Pst* DC3000 in comparison to Mock. Thus, all log_2_ fold change of compounds in mock treatment was 0 (fold change = 1) as shown in the first column. **(D–H)** Relative peak intensities (mean ± SEM, *n* = 4) of five metabolites (aspartic acid, GABA, ethanolamine, fumaric acid, and rutin) identified in the ^1^H NMR spectra that significantly differed among treatments. Different letters indicate significant differences among treatments tested by Mann–Whitney *U* test, *P* < 0.05.

A total of 244 signals were obtained from ^1^H NMR measurement of the mock-, COR-, and *Pst* DC3000-treated tomato plants. The multivariate PLS-DA analysis of the NMR signal profiles resulted in a model with five latent variables (LVs) that cumulatively explained 74.6% of the total metabolomic variation and 91.1% of the elicitor agent treatments, with a 40.7% total model predictability (model statistics: *R*^2^*X* = 0.746, *R*^2^*Y* = 0.911, and *Q*^2^ = 0.407) (**Figure 4**). The first LV explained 23.3% of the variance and separated mock – from both COR – and *Pst* DC3000*-*treated plants (**Figure 4A**). The second LV explained 15.8% and separated COR – from *Pst* DC3000*-*treated plants. The discriminated patterns among mock-, COR-, and *Pst* DC3000*-*treated plants were mainly explained by 80 signals with VIP scores higher than 1 (**Figure 4B** and **Supplementary Figure S3**). Among these 80 NMR signals, 22 were identified, which corresponded to 16 different compounds (**Figure 4C**), including isoleucine (δ 0.96), leucine (δ 1.00), valine (δ 1.04), alanine (δ 1.48), acetate (δ 1.92), glutamate (δ 2.04), malic acid (δ 2.48), aspartic acid (δ 2.64, 2.68, 2.80), citric acid (δ 2.72), gamma-aminobutyric acid (GABA, δ 3.00), ethanolamine (δ 3.12), sucrose (δ 5.40), chlorogenic acid (δ 6.40, 6.44, 6.88), rutin (δ 6.52, 7.00), fumaric acid (δ 6.56), and phenylalanine (δ 7.56). Both COR and *Pst* DC3000 treatments significantly increased leaf content of aspartic acid, ethanolamine and fumaric acid. However, increased GABA and rutin levels were only observed in *Pst* DC3000*-*treated plants (**Figures 4D–H**). For the other identified compounds, we did not find significant differences among treatments.

### *Pst* DC3000-Derived Medium Enhances Tomato Resistance Against WFT

**FIGURE 5:**
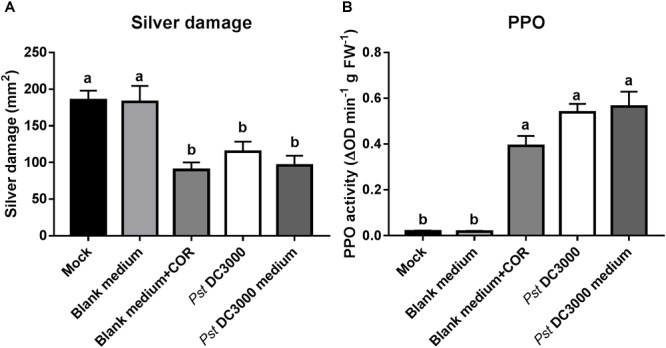
Effect of *Pst* DC3000-derived medium on tomato resistance against WFT and JA-associated responses. **(A)** Silver damage symptoms (mean ± SEM, *n* = 10) in tomato plants infiltrated with mock solution (mock), blank medium, 0.68 μM coronatine (COR) dissolved in blank medium (blank medium + COR), *Pseudomonas syringae* pv. tomato DC3000 (*Pst* DC3000) suspension, or *Pst* DC3000-derived medium (containing 0.68 μM of COR). Plants were infested with western flower thrips (WFT) at 7 days after the initial treatment and evaluated 7 days after WFT infestation. **(B)** Polyphenol oxidase (PPO) activity (mean ± SEM, *n* = 5) was measured in the second leaf from the bottom of tomato plants pressure infiltrated with the above described treatments at 7 days after the initial treatment. Different letters indicate significant differences among treatments tested by Fisher’s LSD test at *P* < 0.05.

To confirm that *Pst* DC3000*-*derived compounds are responsible for the induced resistance against WFT in tomato, we infiltrated plants with the *Pst* DC3000*-*derived medium (containing COR) but no viable bacteria, and compared the effect on tomato resistance against WFT with those triggered by the infiltration with water mock, blank medium control, blank medium + COR, and *Pst* DC3000. Silver damage symptoms were significantly reduced in tomato plants infiltrated with COR, *Pst* DC3000-, or *Pst* DC3000-derived medium compared to water mock or blank medium-treated plants (ANOVA: *P* < 0.05, **Figure 5A**). This reduction in silver damage was stronger in infiltrated leaves, when compared to systemic leaves (i.e., non-infiltrated leaves) (**Supplementary Figure S4**). In addition, PPO activity was induced in blank medium + COR-, *Pst* DC3000-, and *Pst* DC3000*-*derived medium-treated tomato plants compared to water mock and blank medium controls (ANOVA: *P* < 0.05, **Figure 5B**). This confirms the role of COR on the induction of tomato defenses against WFT, and that no bacterial infection is required to elicit WFT resistance.

### Existence of Other Defense Elicitors Besides COR Existed in *Pst* DC3000-Derived Medium

**FIGURE 6:**
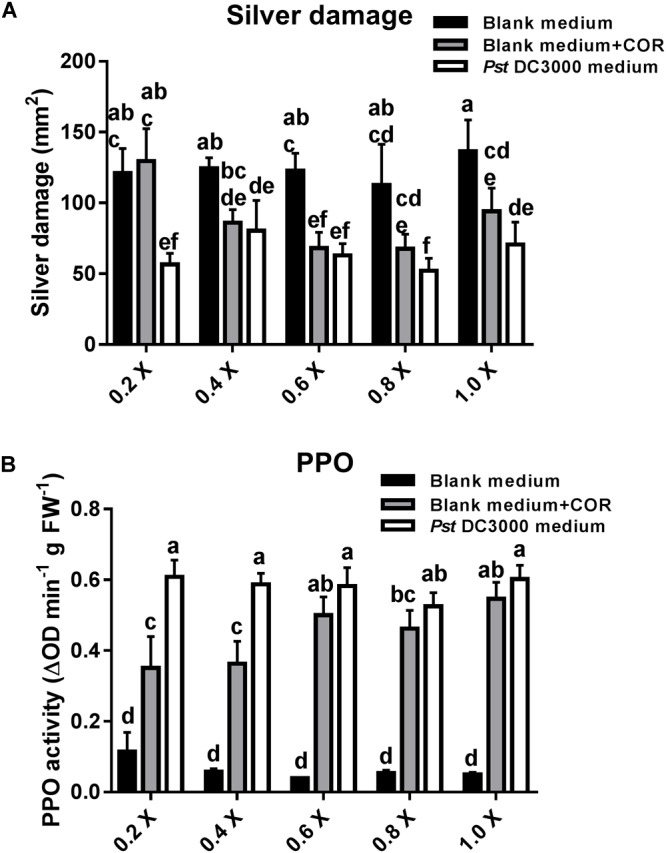
Effect of different concentrations of COR in *Pst* DC3000-derived medium on WFT resistance and JA-associated responses. **(A)** Silver damage symptoms (mean ± SEM, *n* = 7) in tomato plants infiltrated with 0.2, 0.4, 0.6, 0.8, or 1.0× concentrations of (1) blank medium, (2) blank medium + coronatine (COR) (0.64 μM), or (3) *Pseudomonas syringae* pv. tomato DC3000 (*Pst* DC3000)-derived medium (no viable bacteria, containing 0.64 μM of COR). Plants were infested with western flower thrips (WFT) at 7 days after the initial treatment and evaluated 7 days after WFT infestation. **(B)** Polyphenol oxidase (PPO) activity (mean ± SEM, *n* = 5) was measured in the second leaf from the bottom of plants infiltrated with the above described treatments at 7 days after the initial treatment. Different letters indicate significant differences among treatments tested by Fisher’s LSD test at *P* < 0.05.

In the previous experiment, plants infiltrated with COR or *Pst* DC3000-derived medium (containing 0.68 μM COR as in the COR treatments) showed a similar reduction in silver damage symptoms. Yet, the effect of other defense elicitors present in *Pst* DC3000-derived medium might have been masked by the high concentration of COR in the medium. Thus, we further assessed the effect of serial dilutions of blank medium, blank medium + COR, and *Pst* DC3000-derived medium on tomato resistance against WFT and PPO induction (**Figure 6A**). *Pst* DC3000-derived medium- and blank + COR-treated plants showed a significant reduction in silver damage symptoms (GML: *P* < 0.05 for treatment; *P* = 0.078 for dilution; *P* = 0.383 for the interaction). Notably, a stronger reduction in silver damage symptoms was observed in tomato plants treated with 0.2× concentration of *Pst* DC3000-derived medium when compared to 0.2× blank medium and 0.2× blank medium + COR. As these differences were only found at 0.2× concentration, this might explain why the interaction factor between treatment and dilution was not statistically significant. These results suggest that there might be other plant defense elicitors in *Pst* DC3000-derived medium that, maybe in combination with COR, trigger stronger plant defense responses against WFT than COR alone (i.e., in blank medium + COR treatment). Indeed, at 0.2× concentration, induction of the PPO activity was significantly higher in *Pst* DC3000-derived medium-treated plants than in those infiltrated with blank medium + COR (GLM: *P* < 0.05 for interaction) (**Figure 6B** and **Supplementary Table S1**). No significant differences in PPO activity between *Pst* DC3000-derived medium- and blank + COR-treated plants were observed at 0.6, 0.8, and 1.0× concentration.

### Confirmation of the Existence of Other Defense Elicitors in *Pst* DC3000-Derived Medium

**FIGURE 7:**
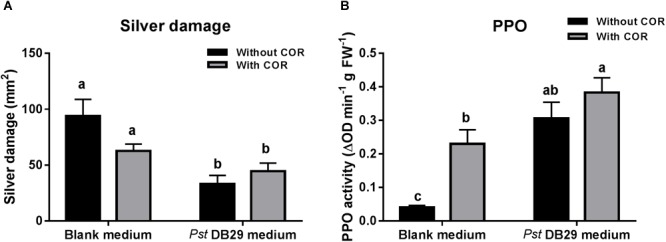
Effect of COR and *Pst* DB29 medium on WFT resistance and JA-associated responses. **(A)** Silver damage symptoms (mean ± SEM, *n* = 10) determined in tomato plants infiltrated with blank medium, 0.14 μM coronatine (COR) in blank medium, *Pseudomonas syringae* pv. tomato DB29 (*Pst* DB29)-derived medium diluted five fold with blank medium or 0.14 μM COR in *Pst* DB29-derived medium diluted five fold with blank medium. Plants were infested with western flower thrips (WFT) at 7 days after the initial treatment and evaluated 7 days after WFT infestation. **(B)** Polyphenol oxidase (PPO) activity (mean ± SEM, *n* = 5) was measured in the second leaf from the bottom of tomato plants infiltrated with the above described treatments at 7 days after the initial treatment. Different letters indicate significant differences among treatments tested by Fisher’s LSD test at *P* < 0.05.

Our previous results showed that while the effect of COR on tomato defenses against WFT was concentration-dependent, the effect of the *Pst* DC3000-derived medium was not, thus pointing out to the existence of other defense elicitors in *Pst* DC3000-derived medium. To further investigate this, we tested the effect of medium obtained from a COR defective mutant of *Pst* DC3000 (*Pst* DB29), blank medium, or both treatments supplemented with a low concentration of COR (0.14 μM) on WFT resistance and PPO activity (**Figure 7**). Silver damage symptoms did not significantly differ between plants infiltrated with blank medium and blank medium + COR (GLM: *P* = 0.994, for COR treatment) (**Figure 7A**), thus confirming our previous results. Yet, a small reduction in silver damage was observed in the infiltrated leaves of blank medium + COR (**Supplementary Figure S5**). Infiltration of plants with *Pst* DB29-derived medium without COR, however, significantly reduced silver damage symptoms when compared to blank medium and blank medium + COR treatments (GLM: *P* < 0.05 for the *Pst* DB29-derived medium; *P* < 0.05 for the interaction). This reduction was significant in both infiltrated and non-infiltrated leaves (**Supplementary Figure S5**). PPO activity was significantly induced by COR and *Pst* DB29-derived medium (GLM: *P* < 0.05 for COR treatment; *P* < 0.05 for the *Pst* DB29-derived medium). Furthermore, *Pst* DB29 + COR-treated plants showed a slight higher PPO induction when compared to *Pst* DB29-derived medium and blank medium + COR treatments (GLM: *P* = 0.104 for their interaction) (**Figure 7B**).

## Discussion

Activation of defense-associated signaling pathways by using natural or synthetic defense elicitors has shown to increase plant resistance against different arthropod herbivores, and it might be regarded as a valuable strategy for pest control in agriculture (Thaler, 1999) in combination with other IPM techniques, such as biological control. Here, we have shown that infiltration with COR, *Pst* DC3000, or *Pst*-derived medium increased tomato resistance against WFT through the induction of enzymatic and chemical defenses.

Our results first showed that infiltration of tomato plants with the COR-producing bacteria *Pst* DC3000 or COR alone significantly reduced WFT-associated damage in non-choice whole plant bioassays (**Figure 1**). This is in line with previous reports. Cui et al. (2005) described that the increased susceptibility to the caterpillar *Trichoplusia ni* in *Arabidopsis* plants infiltrated with virulent strains of *P. syringae* ES4326 was counteracted by COR, and that COR alone increased *Arabidopsis* resistance to this caterpillar. In tomato, Stout et al. (1999) described that infiltration with *P. syringae* pv. tomato significantly reduced *Helicoverpa Zea* larvae growth. Here we report on the effects of both *Pst* DC3000 and COR infiltration on tomato resistance against WFT. Furthermore, we show that not only COR, but that presence of other defense elicitors in *Pst*-derived medium can increase tomato resistance to WFT.

The enhancement of plant defenses against arthropod herbivores by infiltration of COR-producing *Pst* or COR itself has been explained by the strong induction of the JA-associated defense signaling pathway and suppression of the SA defense signaling (Cui et al., 2005). Analysis of the effect of COR and *Pst* DC3000 infiltration on the activation of JA and SA signaling pathways showed that both treatments strongly induced the expression of the JA-associated gene marker *WIPI-II*, which encodes for a proteinase inhibitor II protein (PI-II), and increased activity of the JA-related defensive enzyme PPO at 7 days after the infiltration. This agrees with previous results described by Stout et al. (1999), who found that *Pst* infiltration increased PI-II and PPO activities in infiltrated tomato plants. *Pst* DC3000 is reported to activate JA signaling in tomato (Zhao et al., 2003; Uppalapati et al., 2005) and *Arabidopsis* (He et al., 2004), which is proposed to be explained by the action of *Pst* DC3000-derived COR and type III effectors (He et al., 2004). Our results showed that application of COR also induced the expression of the SA-associated gene marker *PR-P6*, a pathogen defense-related gene (PR) (**Figure 2C**). Yet, the magnitude of the induction of *PR-P6* was approximately 30 times lower than that of *WIPI-II* in COR-treated plants. Both COR treatment and infection with *Pst* DC3000 lead to slight increases of SA levels in *Arabidopsis* (Uppalapati et al., 2005) and tomato (Zhao et al., 2003). In tomato, this induction has been described to be stronger in COR deficient *Pst*, and thus it was suggested to be highly suppressed by the activation of JA signaling in COR-producing *Pst* (Zhao et al., 2003). The lack of induction of *PR-P6* in *Pst* DC3000-infected tomato plants (**Figure 2B**) might be explained by our sampling time for gene expression analysis. Hence, induction of PRs has been generally observed 24 h after *Pst* infiltration (Zhao et al., 2003; Uppalapati et al., 2008; López-Gresa et al., 2011). Overall, the strong activation of JA-associated defenses by *Pst* DC3000 and COR infiltration might explain the increased tomato resistance against WFT. Previous studies have shown that induction of JA defenses can reduce WFT-associated damage in tomato and other plant species (Li et al., 2002; Abe et al., 2009; Escobar-Bravo et al., 2017). Activation of JA defense signaling is often associated with reduced plant growth (Guo et al., 2018). Interestingly, our results showed that neither COR or *Pst* DC3000 infiltration significantly affected plant dry biomass or height of tomato plants (**Supplementary Figure S6**). Additionally, we did not detect any *Pst* DC3000 colonies in systemic leaves of *Pst* DC3000-infiltrated plants, but only in the local leaves (**Supplementary Figure S7**), confirming that even localized *Pst* DC3000 infections have a great impact on tomato defenses against other biotic stressors.

Activation of JA signaling pathway through exogenous application of jasmonates, such as the volatile form of JA methyl jasmonate (MeJA) (Boughton et al., 2005; Maes and Goossens, 2010; Tian et al., 2012; Escobar-Bravo et al., 2017) is reported to increase type VI glandular trichome density in tomato leaves. We thus hypothesized that infiltration with *Pst* DC3000 or COR might induce these tomato defenses as well. Our results, however, showed that none of these treatments increased type VI trichome densities in newly formed leaves at 7 days after infiltration. This might be explained by differences in the magnitude of the induction of JA defenses when plants are treated with exogenous application of COR or *Pst* DC3000 infiltration, but also by the activation of different defense signaling pathways. Hence, although COR and MeJA application shared similar activities on tomato plants, some sets of genes are differently regulated by these two compounds (Uppalapati et al., 2005; Tsai et al., 2011). Both COR and *Pst* DC3000 are reported to induce JA, ET, and auxin signaling pathways (O’donnell et al., 2003; Cohn and Martin, 2005; Uppalapati et al., 2005), and COR slightly induced SA signaling as well. Whether the induction of these signaling pathways explains the lack of induction of trichomes in COR and *Pst* DC3000 infiltrated plants would require further research. Alternatively, COR-mediated activation of SA signaling might have attenuated JA-mediated induction of trichomes (Traw and Bergelson, 2003), as both signaling are known to interact via antagonistic crosstalk (Pieterse et al., 2012). Together, these results suggest that COR- and *Pst-*DC3000-mediated induction of tomato resistance against WFT is not explained by increased type-VI trichome densities.

An untargeted metabolomic analysis of tomato leaves infiltrated with COR or *Pst* DC3000 revealed that both treatments induced similar but not the same metabolomic changes. Both COR and *Pst* DC3000 increased the leaf content of organic acids, phenolics, and amino acids (**Figure 4**). These results are in agreement with those reported by López-Gresa et al. (2010, 2011), where higher concentrations of amino acids, organic acids, rutin, and phenylpropanoids were detected in *Pst*-infected tomato plants. However, no comparison between the effects of COR and *Pst* infiltration on plant metabolome has been performed before. Interestingly, our results showed that the levels of the amino acid aspartic acid and the non-protein amino acid GABA, as well as the phenolic rutin, were slightly higher in *Pst* DC3000-infiltrated tomato leaves. Yet, these differences did not affect the levels of resistance of tomato plants against WFT, as both COR and *Pst* DC3000 significantly reduced silver damage symptoms in infiltrated plants (**Figure 1**). The increase in some of these compounds might have influenced tomato defenses against WFT. For instance, high concentrations of the flavonoid rutin (quercetin-3-O-β-rutinoside) have been reported to deter herbivore feeding (reviewed by Simmonds, 2001). On the other hand, increases in GABA levels are reported to occur in *Pst* DC3000-infected plants (Ward et al., 2010), but also in response to other biotic and abiotic stresses (Bouché et al., 2003). Although all the functions of GABA in plants have not been completely elucidated, it is induced upon herbivory or insects crawling on the leaf surface (Bown et al., 2002; Scholz et al., 2015), and it has a negative effect on arthropod’s performance when ingested by feeding in transgenic plants with elevated GABA levels or in GABA-enriched artificial diets (Ramputh and Bown, 1996; McLean et al., 2003; Scholz et al., 2015). Yet, whether its induction might affect tomato resistance against WFT would need further research.

Our results further showed that application of *Pst* DC3000-derived medium (without viable *Pst* bacteria and containing 0.68 μM of COR), COR (0.68 μM), or *Pst* DC3000, all increased tomato resistance against WFT (**Figure 5A**). Moreover, these treatments increased PPO activities in infiltrated leaves, indicating the activation of JA signaling (**Figure 5B**). Hence, infiltration of tomato plants with ca. seven times less COR than in our initial experiments (i.e., 5 μM, see **Figure 1**) resulted in a similar reduction in silver damage symptoms. This suggested that COR has a strong impact on tomato defenses even at low concentrations, and that we might have overlooked the possible effect of other defense elicitors present in *Pst* DC3000-derived medium. This prompted us to further investigate whether infiltration with much lower concentrations of COR alone or in *Pst* DC3000-derived medium had the same effects on tomato resistance against WFT. Notably, infiltration of tomato plants with a 0.2× concentration of *Pst* DC3000-derived medium (containing 0.14 μM of COR) resulted in a stronger reduction of silver damage symptoms than application of COR (0.14 μM) alone dissolved in blank medium. Moreover, induction of PPO activity was higher in plants infiltrated with a 0.2× concentration of *Pst* DC3000-derived medium than with a 0.2× concentration of COR or blank medium. Hence, this suggests that the presence of other defense elicitors in *Pst* DC3000-derived medium might increase tomato resistance against WFT, and that this induction is also probably explained by a stronger activation of JA signaling. Indeed, further assays using a COR deficient mutant of *Pst* DC3000, *Pst* DB29, showed that tomato plants infiltrated with *Pst* DB29-derived medium displayed lower silver damage symptoms after WFT infestation and induced PPO activities as well (**Figures 7A,B**). It should be noted that *Pst* DB29 is defective in the synthesis of COR precursors, CFA and CMA, both reported to induce some JA/wound associated plant responses in tomato, but at much less magnitude than COR (Uppalapati et al., 2005). Thus, activation of tomato defenses against WFT could not be explained by the presence of CFA and CMA in the *Pst* DB29-derived medium. We hypothesize that these responses might be explained by changes in the culture medium composition in terms of (1) primary or secondary metabolites modified in the medium by the *Pst* growth, or (2) presence of *Pst*-derived effectors. For instance, He et al. (2004) described that effectors secreted by the type III secretion system of *Pst* DC3000 can augment the JA-signaling pathway to promote virulence. Nevertheless, this requires further research.

In summary, our study shows that infiltration with COR and *Pst* DC3000 increases tomato resistance against WFT by activating JA-associated defenses, but not type-VI leaf trichome densities. Our results also show that *Pst* DC3000-derived medium contains other defense elicitors that can increase resistance against WFT in infiltrated tomato plants, thus providing a potential treatment for WFT control in agriculture systems.

## Author Contributions

GC, RE-B, PK, and KL carried out the experimental design. GC conducted the infiltration, insect experiments, and chemical analyses. GC and RE-B performed the RT-qPCRs. GC, RE-B, PK, and KL performed the data analysis and interpretation. HK interpreted the NMR data. GC wrote the draft manuscript. All authors critically reviewed and approved the final version of the manuscript.

## Conflict of Interest Statement

The authors declare that the research was conducted in the absence of any commercial or financial relationships that could be construed as a potential conflict of interest.

## Footnotes

1 Escobar-Bravo, R., Ruijgrok, J., Kim, H. K., Grosser, K., Van Dam, N. M., Klinkhamer, P. G. L., et al. (in press). Light intensity-mediated induction of trichome-associated allelochemicals increases resistance against thrips in tomato. *Plant Cell Physiol.* doi: 10.1093/pcp/pcy166

2 http://fiji.sc/Fiji
