# Supplementary material for: Induced Resistance Against Western Flower Thrips by the Pseudomonas syringae-Derived Defense Elicitors in Tomato
Source: Front Plant Sci. 2018 Sep 26;9:1417. doi: 10.3389/fpls.2018.01417 (PMC6182256; doi:10.3389/fpls.2018.01417)
Supplement: Supplementary file 1 [file Table_1.docx]

***Supplementary materials***

**Induced resistance against western flower thrips by the *Pseudomonas syringae*-derived defense elicitors in tomato**

**Gang Chen^1*^, Rocío Escobar-Bravo^1^, Hye Kyong Kim^1^, Kirsten A. Leiss^2^, Peter G.L. Klinkhamer^1^**

^1^Plant Science and Natural Products, Institute of Biology, Leiden University, The Netherlands

^2^Business Unit Horticulture, Wageningen University and Research Centre, Bleiswijk, The Netherlands

***Correspondence**

Gang Chen

Email: [g.chen@biology.leidenuniv.nl](mailto:g.chen@biology.leidenuniv.nl)

# **1 Supplementary Methods**

## **1.1 Supplementary Method 1 Gene expression analysis**

The gene-specific primers used for the RT-qPCRs are listed below.

***Wound inducible proteinase inhibitor II* (Solyc01g095200)**

WIPI II_F: 5´- GACAAGGTACTAGTAATCAATTATCC -3´

WIPI II_R: 5´- GGGCATATCCCGAACCAAGA -3´

***Pathogenesis related-protein 6* (Solyc00g174340)**

PR-P6_F: 5´- GTA CTG CAT CTT CTT GTT TCC A -3´

PR-P6_R: 5´- TAG ATAAGT GCT TGA TGT GCC -3´

***Actin* (Solyc03g078400)**

SlActin_F: 5´- TTAGCACCTTCCAGCAGATGT -3´

SlActin_R: 5´- AACAGACAGGACACTCGCACT -3´

# **2 Supplementary Figures and Tables**

2.1 Supplementary Figures

**
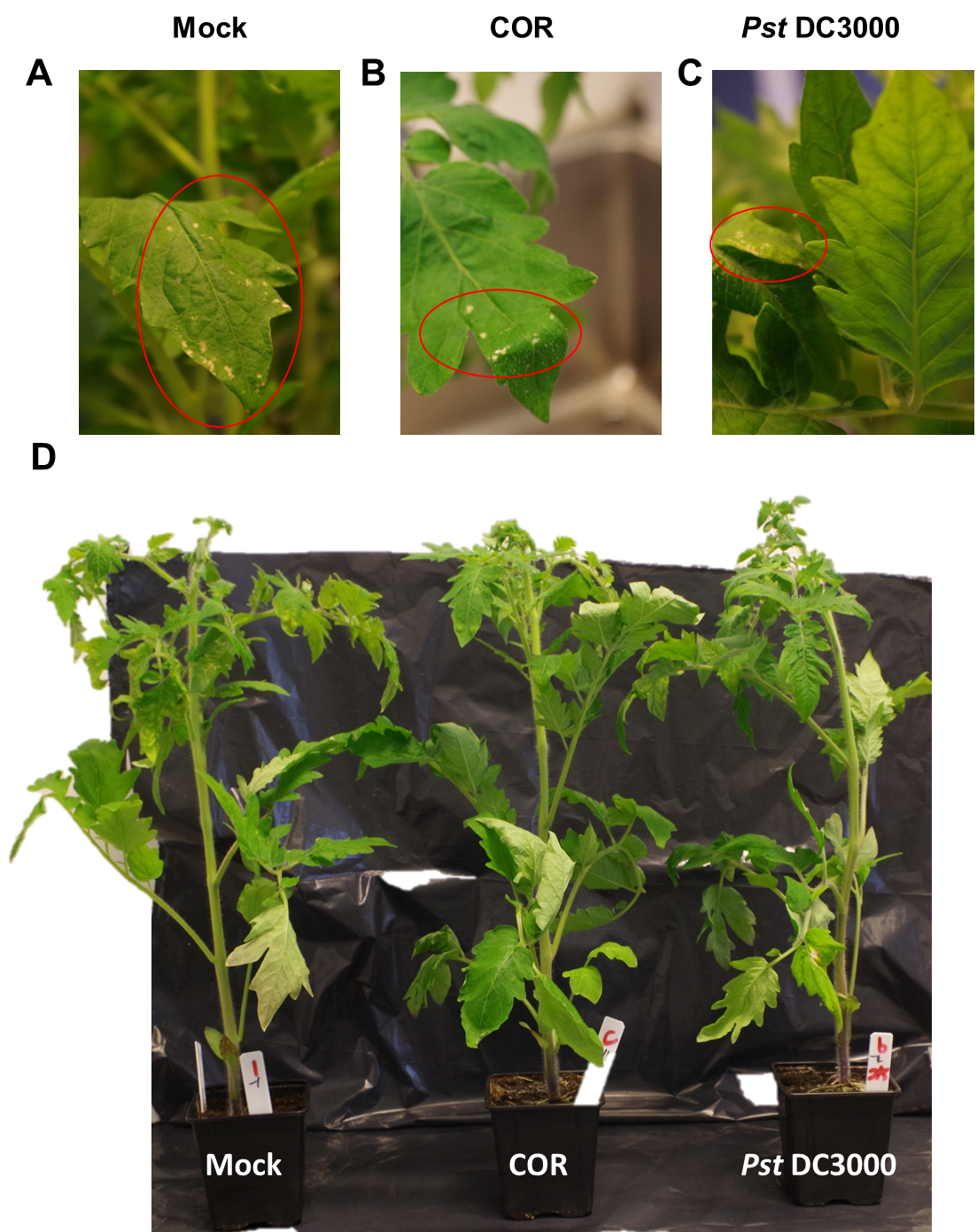
**

**Supplementary Figure S1.** Representative photographs of leaves from thrips-infested (A) Mock-, (B) coronatine (COR)- or (C) *Pseudomonas syringae* pv. tomato DC3000 (*Pst* DC 3000)-treated plants. The Red circles indicate the silver damage symptoms caused by western flower thrips (WFT) feeding. (D) Representative photographs of the WFT-infested plants subjected to mock-, COR- or *Pst* DC3000 treatments. Four leaflets of four-week old tomato plants were infiltrated with mock solution (water), 5 µM COR solution or 10^8^ cfu ml^-1^ *Pst* DC3000. Seven days after treatments plants were subjected to non-choice whole plant thrips bioassays. Silver damage symptoms were determined at 7 days after infestation.

**Supplementary Figure S2. Effect of COR and *Pst* DC3000 on tomato resistance against WFT.** Silver damage symptoms (mean ± SEM, *n* = 15) were determined in (**A**) infiltrated and (**B**) non-infiltrated leaves of mock-, coronatine (COR)- and *Pseudomonas syringae* pv. tomato DC3000 *(Pst* DC3000)-treated tomato plants. Plants were infested with (western flower thrips) WFT at 7 days after the initial treatment and evaluated 7 days after WFT infestation. Different letters indicate significant differences among treatments tested by Fisher’s LSD test at *P* < 0.05.

**B**

**A**

**Supplementary Figure S3. Important NMR signals that contributed to the metabolome differentiation among treatments.** Heatmap of the 78 out of 80 signals detected by NMR and displaying VIP scores > 1 based on PLS-DA analysis. Each heatmap column displays the log_2_ fold change of relative peak intensity of the compounds differentially induced in Mock, COR or *Pst* DC3000 samples in comparison to Mock. Log_2_ fold change of compounds in the mock treatment was 0 (fold change = 1). The other 2 out of 80 NMR signals cannot be shown in the Heatmap, because the mean relative peak intensity of Mock was 0.

**B**

**A**

**Supplementary Figure S4.** **Effect of *Pst* DC3000-derived medium on tomato resistance against WFT.** Silver damage symptoms (mean ± SEM, *n* = 10) determined in (**A**) infiltrated and (**B**) non-infiltrated leaves of tomato plants treated with a mock solution (mock), blank medium, 0.68 µM coronatine (COR) dissolved in blank medium (blank medium + COR), *Pseudomonas syringae* pv. tomato DC3000 (*Pst* DC3000) suspension or *Pst* DC3000-derived medium (containing 0.68 µM of COR). Plants were infested with western flower thrips (WFT) at 7 days after the initial treatment and evaluated at 7 days after WFT infestation. Different letters indicate significant differences among treatments tested by Fisher’s LSD test at *P* < 0.05.

**B**

**A**

**Supplemental Figure S5. Effect of COR and *Pst* DB29 medium on WFT resistance.** Silver damage symptoms (mean ± SEM, *n* = 10) determined in (**A**) infiltrated and (**B**) non-infiltrated leaves of tomato plants treated with blank medium, 0.14 μM COR in blank medium, *Pseudomonas syringae* pv. tomato DB29 (*Pst* DB29)-derived medium diluted five-fold with blank medium or 0.14 μM COR in *Pst* DB29-derived medium diluted five-fold with blank medium. Plants were infested with western flower thrips (WFT) at 7 days after the initial treatment and evaluated 7 days after WFT infestation.

**Supplementary Figure S6. Effect of mock solution, COR, or *Pst* DC3000 on plant growth.** (**A**) Dry biomass of the above ground plant material and (**B**) stem height were determined in tomato plants infiltrated with a mock solution (mock), 5 µM of coronatine (COR) or 10^8^ cfu ml^-1^ of *Pseudomonas syringae* pv. tomato DC3000 (*Pst* DC3000) suspension. Measurements were performed at 7 days after the initial treatments. Depicted are the average (± SEM) of fifteen replicates. Different letters indicate significant differences among treatments (One way ANOVA followed by Fisher’s LSD test, *P* < 0.05).


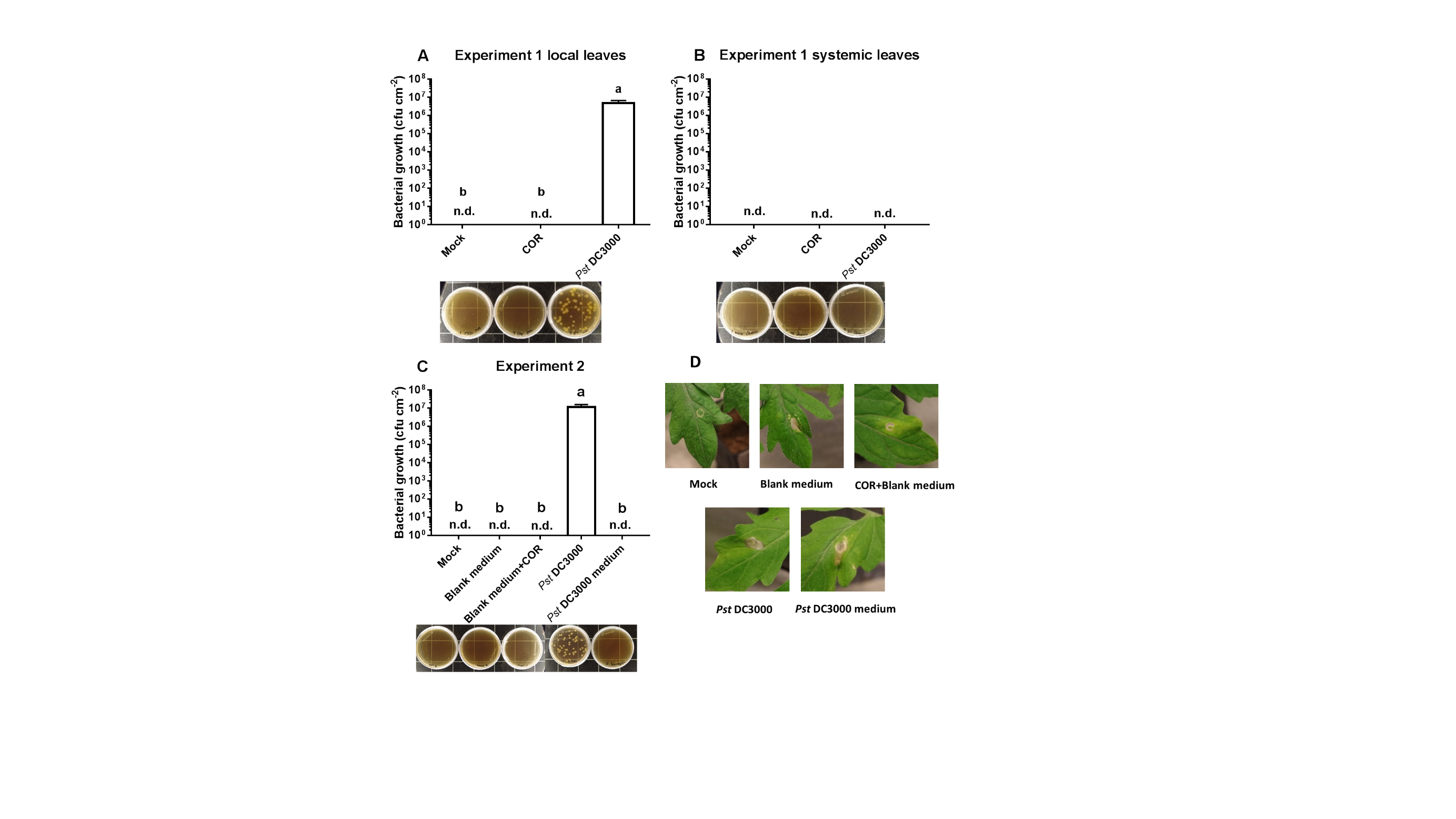


**Supplementary Figure S7. Bacteria growth and symptoms of tomato plants infiltrated with COR, *Pst* DC3000 or *Pst* DC-3000 derived medium.** Four leaflets of four-week old tomato plants were infiltrated with mock solution (water), 5 µM coronatine (COR) solution or 10^8^ cfu ml^-1^ *Pseudomonas syringae* pv. tomato DC3000 (*Pst* DC3000) suspension for experiment 1; or with mock solution (water), blank medium, 0.68 µM COR + blank medium, 10^8^ cfu ml^-1^ *Pst* DC3000 suspension or *Pst* DC3000-derived medium diluted five-fold with blank medium for experiment 2. (**A**) *Pst* DC3000 growth was determined in mock-, COR- and *Pst* DC3000-infiltrated (i.e. local) leaves and (**B**) in non-infiltrated systemic leaves at 7 days after the initial treatments in experiment 1. (**C**) *Pst* DC3000 growth was determined in mock-, blank medium, blank medium +COR, *Pst-*DC3000, and *Pst* DC3000-derived medium-infiltrated (i.e. local) leaves at 7 days after the initial treatment in experiment 2. Differences in bacteria growth among the treatments were tested by non-parametric Kruskal-Wallis rank-sum test followed by Wilcoxon rank-sum test for multiple comparisons. n.d.: not detected. Below each graph, representative photographs of the bacterial colonies visually detected in the 10,000x dilution are shown. **D**) Representative photographs of the symptoms in local leaves observed 7 days after infiltration in the experiment 2 are shown.

**2.2 Supplementary tables**

**Supplementary Table S1. Results of the statistical analysis performed for each figure.**

| **Figure** | **Panel** | **Statistical test** | **Factor and statistic value** | **df** | ***P*** |
| --- | --- | --- | --- | --- | --- |
| **Figure 1** | **None** | One-way ANOVA | COR or *Pst* DC3000; *F* = 8.610 | 2 | *P* = 0.001 |
| **Figure 2** | **A** | One-way ANOVA | COR or *Pst* DC3000; *F* = 25.494 | 2 | *P* < 0.001 |
|  | **B** | One-way ANOVA | COR or *Pst* DC3000; *F* = 8.943 | 2 | *P* = 0.007 |
|  | **C** | One-way ANOVA | COR or *Pst* DC3000; *F* = 7.038 | 2 | *P* = 0.014 |
| **Figure 3** | **A** | One-way ANOVA | COR or *Pst* DC3000; *F* = 2.927 | 2 | *P* = 0.071 |
|  | **B** | One-way ANOVA | COR or *Pst* DC3000; *F* = 3.744 | 2 | *P* = 0.037 |
| **Figure 4** | **D** | Kruskal-Wallis test | COR or *Pst* DC3000; *χ*² = 7.385 | 2 | *P* = 0.025 |
|  | **E** | Kruskal-Wallis test | COR or *Pst* DC3000; *χ*² = 8.000 | 2 | *P* = 0.018 |
|  | **F** | Kruskal-Wallis test | COR or *Pst* DC3000; *χ*² = 7.731 | 2 | *P* = 0.021 |
|  | **G** | Kruskal-Wallis test | COR or *Pst* DC3000; *χ*² = 7.538 | 2 | *P* = 0.023 |
|  | **H** | Kruskal-Wallis test | COR or *Pst* DC3000; *χ*² = 8.769 | 2 | *P* = 0.012 |
| **Figure 5** | **A** | One-way ANOVA | COR, *Pst* DC3000 or *Pst* DC3000 medium; *F* = 9.790 | 4 | *P* < 0.001 |
|  | **B** | One-way ANOVA | COR, *Pst* DC3000 or *Pst* DC3000 medium; *F* = 156.551 | 4 | *P* < 0.001 |
| **Figure 6** | **A** | GLM | Dilution; *Wald χ*² = 8.400 | 4 | *P* = 0.078 |
|  |  |  | COR or *Pst* DC3000 medium; *Wald χ*² = 55.789 | 2 | *P* < 0.001 |
|  |  |  | Interaction; *Wald χ*² = 8.537 | 8 | *P* = 0.383 |
|  | **B** | GLM | Dilution; *Wald χ*² = 4.472 | 4 | *P* = 0.346 |
|  |  |  | COR or *Pst* DC3000 medium; *Wald χ*² = 429.336 | 2 | *P* < 0.001 |
|  |  |  | Interaction; *Wald χ*² = 17.691 | 8 | *P* = 0.024 |
| **Figure 7** | **A** | GLM | *Pst* DB29 medium; *Wald χ*² = 31.481 | 1 | *P* < 0.001 |
|  |  |  | COR; *Wald χ*² < 0.001 | 1 | *P* = 0.994 |
|  |  |  | Interaction; *Wald χ*² = 7.104 | 1 | *P* = 0.008 |
|  | **B** | GLM | *Pst* DB29 medium; *Wald χ*² = 36.311 | 1 | *P* < 0.001 |
|  |  |  | COR; *Wald χ*² = 14.623 | 1 | *P* < 0.001 |
|  |  |  | Interaction; *Wald χ*² = 2.642 | 1 | *P* = 0.104 |
